# Effects of Dietary Allicin Supplementation on Laying Performance, Egg Quality, Serum Lipid Indices, and Gut Microbiota Composition in Kangle Chickens

**DOI:** 10.3390/ani16203168

**Published:** 2026-10-09

**Authors:** Qingcan Fan, Jinsheng Yang, Manyan Chen, Wenbo Yan, Haigen Li, Lucheng Zheng, Wei Hu, Hu Yang, Ben Liu, Qianqian Wang

**Affiliations:** 1 College of Life Sciences and Resource Environment, Yichun University, Yichun 336000, China; 999341@jxycu.edu.cn (Q.F.);; 2 Institute of Animal Science, Yichun Academy of Sciences, Yichun 336000, China

**Keywords:** phytogenic feed additive, yolk cholesterol, egg shape index, average egg weight, duodenal microbiota

## Abstract

**Simple Summary:**

Allicin, a natural bioactive component of garlic, exhibits antibacterial, antioxidant, and lipid-regulating activities and has attracted considerable interest in poultry nutrition. However, studies investigating its effects on indigenous Chinese laying hens remain scarce. This study evaluated the effects of dietary allicin supplementation at 0 (control), 200, 400, and 600 mg/kg on laying performance, egg quality, serum lipid indices, and gut microbiota in Kangle chickens. The results showed that supplementation with 400 mg/kg allicin significantly increased average egg weight (AEW) and improved egg shape index (ESI), while reducing serum total cholesterol (TC) and yolk total cholesterol (YTC) levels. In addition, dietary allicin supplementation at 600 mg/kg was associated with alterations in gut microbial composition and shifts in predicted metabolic functions. These findings suggest that allicin may serve as a promising natural feed additive for increasing egg weight, improving egg shape index, reducing serum and yolk cholesterol levels, and modulating gut microbiota in Kangle laying hens.

**Abstract:**

Allicin was supplemented into the diet of Kangle chickens, a native Chinese chicken breed, and its effects on laying performance, egg quality, serum lipid indices, and gut microbiota were evaluated. A total of 144 hens at 30 weeks of age with similar body weights were randomly assigned to four groups (CON, AL200, AL400, and AL600), which received a basal diet supplemented with 0 (control), 200, 400, and 600 mg/kg allicin, respectively. The experiment consisted of a 4-day adaptation period followed by a 28-day experimental period. The results showed that, compared with the CON group, average egg weight (AEW) was increased in the AL200 and AL400 groups (*p* < 0.05), whereas average daily feed intake (ADFI) was reduced in the AL600 group (*p* < 0.05). Egg shape index (ESI) was increased in all allicin-supplemented groups relative to the CON group (*p* < 0.05). Regarding lipid profiles, serum total cholesterol (TC) levels were decreased in all allicin-supplemented groups (*p* < 0.05); serum triglyceride (TG) and low-density lipoprotein cholesterol (LDL-C) concentrations were lower in the AL600 group (*p* < 0.05); and yolk total cholesterol (YTC) was significantly reduced in the AL400 and AL600 groups (*p* < 0.05). In addition, the AL600 group had a significantly higher duodenal index (*p* < 0.05), whereas abdominal fat percentage (AFP) was decreased compared with the CON group (*p* < 0.05). Gut microbiota analysis revealed that the AL600 group showed significant alterations in alpha diversity indices (*p* < 0.05). The relative abundance of potentially beneficial bacterial taxa, such as *Oscillospiraceae* and *Romboutsia_B*, was significantly increased in this group (*p* < 0.05). Collectively, these findings suggest that allicin may serve as a promising natural feed additive for increasing egg weight and egg shape index, reducing serum and yolk cholesterol levels, and modulating gut microbiota and predicted metabolic functions in Kangle laying hens.

## 1. Introduction

Plant-derived feed additives have attracted increasing attention because of their environmentally friendly nature and high efficacy. Allicin, a unique organosulfur compound derived from garlic, is responsible for the characteristic pungent odor and irritant properties of garlic and is chemically known as diallyl thiosulfinate [1]. Intact garlic contains alliin, the precursor of allicin, rather than free allicin [2]. Upon cell disruption, alliinase is released from vacuoles and hydrolyzes alliin to allyl sulfenic acid; two molecules of this intermediate then spontaneously dimerize to form one molecule of allicin [3]. After entering the body, allicin rapidly reacts with glutathione (GSH) to form S-allyl mercaptoglutathione, whereas free allicin diffuses across cell membranes and induces S-allyl oxidative modification of thiol groups in protein cysteine residues [4]. The reversible oxidation and reduction of thiol groups are central to the bioactivity of allicin, enabling the regulation of multiple cellular processes and contributing to its antioxidant [5], immunomodulatory [6], and lipid-regulatory [7] effects.

Allicin and its derivatives have been reported to exert multiple beneficial effects in poultry production. For example, dietary supplementation with 7.5 g/kg garlic powder, whose main active constituents are allicin and its derivatives, significantly improved body weight gain and feed conversion ratio (FCR) in broilers and reduced serum triglyceride (TG) and low-density lipoprotein cholesterol (LDL-C) levels; it also suppressed the expression of 3-hydroxy-3-methylglutaryl-CoA reductase (HMGCR) and fatty acid synthase (FAS), which are key enzymes in lipid synthesis [8]. Similarly, dietary inclusion of 200 mg/kg bioactive garlic oil, with allicin derivatives as its main active constituents, significantly increased body weight, decreased TG and very low-density lipoprotein (VLDL) levels, and reduced the abundance of potentially harmful intestinal bacteria, thereby helping to maintain gut microbial homeostasis [9]. Supplementation with 300 mg/kg allicin significantly enhanced body weight, regulated glucose homeostasis, and promoted skeletal muscle glucose uptake in broilers [10]. Moreover, allicin and its derivatives have been shown to modulate gut microbiota in animals. In mice fed a high-fat diet, allicin treatment significantly increased gut microbial alpha diversity [11] and the abundance of *Bifidobacterium* and *Lactobacillus* [12]. However, studies on the effects of allicin on laying performance and gut microbial composition in Chinese indigenous laying hens during the laying period remain limited.

The Kangle chicken (*Gallus gallus domesticus*), also known as Wanzai Sanhuang chicken, is an indigenous chicken breed native to Jiangxi Province, China. This breed is favored by consumers for its desirable characteristics, including excellent meat quality and strong disease resistance. Therefore, the present study was designed to investigate the effects of dietary allicin supplementation at graded levels on laying performance, serum lipid indices, and duodenal microbiota composition in Kangle chickens, aiming to provide a scientific basis for the application of allicin in indigenous poultry production.

## 2. Materials and Methods

### 2.1. Experimental Animals and Treatments

All experimental procedures were approved by the Animal Ethics Committee of Yichun University (Approval No. 2024057). A total of 144 thirty-week-old Kangle laying hens with similar body weight and laying rate were obtained from the breeding facility at Yichun University and randomly assigned to four groups (six replicates per group, with six hens per replicate). The control group (CON) received a basal diet without allicin, whereas the AL200, AL400, and AL600 groups received the same basal diet supplemented with 200, 400, and 600 mg/kg allicin, respectively [13]. The basal diet was formulated as mash feed according to the Chinese agricultural industry standard NY/T 3645-2020 [14] (Nutrient Requirements of Yellow-Feathered Broilers), and its composition and nutrient content are presented in Table 1. Allicin (purity ≥ 98%) was purchased from Shanghai Yuanye Bio-Technology Co., Ltd. (Shanghai, China; Cat. No. S25256; Lot No. 22IS226547).

For the allicin-treated groups, an aqueous allicin emulsion was thoroughly mixed with the feed at the designated concentration, whereas the control group received an equal volume of water mixed with the feed following the same procedure. A fresh emulsion was prepared before each feeding. During preparation, the emulsion was first premixed with a small portion of the feed. The mixture was then sealed and left to stand for 30 min, after which it was mixed with the remaining feed to minimize allicin volatilization.

The experiment lasted for 32 days, comprising a 4-day adaptation period followed by a 28-day experimental period. Feed and water were available ad libitum. Feed was replenished three times per day (morning, noon, and evening), and residual feed was collected and weighed each morning before feeding. The lighting program consisted of natural light supplemented with artificial light to provide a total of 16 h of light per day at an intensity of at least 10 lx. Manure was removed regularly to maintain hygienic conditions in the henhouse.

### 2.2. Sample Collection and Processing

At the end of the 28-day experimental period, two hens per replicate were randomly selected, and blood samples were collected from the wing vein into vacuum tubes without anticoagulant. Within 30 min of collection, samples were centrifuged at 1500× *g* at 4 °C for 10 min. The serum was then carefully collected, aliquoted, and stored at −80 °C for subsequent biochemical analyses. In addition, two hens per replicate from the CON and AL600 groups were randomly selected and slaughtered, and body weight was recorded immediately before the procedure. The liver, duodenum, jejunum, ovary, and abdominal fat were immediately excised and weighed to calculate the corresponding organ indices. The duodenal luminal contents were collected, snap-frozen in liquid nitrogen, and stored at −80 °C until analysis.

### 2.3. Measurement of Indicators

#### 2.3.1. Production Performance

During the 28-day experimental period, the number of eggs laid, egg weight, and feed consumption were recorded daily for each replicate. At the end of the experimental period, the average laying rate (ALR), average daily feed intake (ADFI), average egg weight (AEW), and FCR were calculated for each replicate. Organ indices, including the liver index, duodenal index, jejunum index, and ovary index, as well as abdominal fat percentage (AFP), were calculated as organ weight relative to body weight (organ weight/body weight × 100). On the final day of the experiment, three freshly laid eggs were randomly collected from each replicate. The transverse and longitudinal diameters of each egg were measured using a digital vernier caliper, and the egg shape index (ESI) was calculated as (transverse diameter/longitudinal diameter) × 100. In addition, egg quality parameters, including yolk ratio (YR), albumen height (AH), yolk color (YC), and Haugh unit (HU), were determined within 24 h after collection using a multifunctional egg quality tester (EA-01; Orka, Herzliya, Israel).

#### 2.3.2. Lipid Metabolism Indices

The concentrations of serum TG, LDL-C, total cholesterol (TC), and high-density lipoprotein cholesterol (HDL-C) were measured using commercial assay kits according to the manufacturer’s instructions, with absorbance read on a multifunctional microplate reader (AF2200 Pro; Eppendorf, Hamburg, Germany). In addition, two additional freshly laid eggs were randomly collected from each replicate, and yolk total cholesterol (YTC) content was determined using a cholesterol assay kit according to the manufacturer’s instructions. All assay kits were purchased from Nanjing Jiancheng Bioengineering Institute (Nanjing, China).

### 2.4. Microbiota 16S rRNA Sequencing of Duodenal Contents

From the six replicates of the CON group and six replicates of the AL600 group, four replicates per group were randomly selected, and one duodenal luminal content sample was randomly selected from each selected replicate, yielding eight samples in total. These samples were sent to Shanghai Personal Biotechnology Co., Ltd. (Shanghai, China) for 16S rRNA gene amplicon sequencing. Nucleic acids were extracted from 0.2–0.5 g of each sample using the OMEGA Soil DNA Kit (Omega Bio-Tek, Norcross, GA, USA), and DNA quantity and quality were assessed using a NanoDrop NC2000 spectrophotometer (Thermo Fisher Scientific, Waltham, MA, USA). The V3–V4 hypervariable region of the bacterial 16S rRNA gene was amplified using the forward primer 5′-ACTCCTACGGGAGGCAGCA-3′ and the reverse primer 5′-GGACTACHVGGGTWTC TAAT-3′, with a 7–10 nt barcode appended to the forward primer for multiplexing. After PCR amplification, the products were checked by agarose gel electrophoresis, and the target fragments were recovered and quantified with the Quant-iT PicoGreen dsDNA Reagent (Invitrogen, Carlsbad, CA, USA) on a microplate reader (BioTek Instruments, Winooski, VT, USA). Sequencing libraries were constructed from 30 ng of DNA per sample using the TruSeq Nano DNA LT Library Prep Kit (Illumina, San Diego, CA, USA). After size selection and purification by agarose gel electrophoresis, library quality was evaluated on an Agilent Bioanalyzer using the Agilent High Sensitivity DNA Kit (Agilent Technologies, Santa Clara, CA, USA) and quantified with the Quant-iT PicoGreen dsDNA Assay Kit (Thermo Fisher Scientific, Waltham, MA, USA) on a Promega QuantiFluor instrument (Promega, Madison, WI, USA). The libraries were then normalized to 2 nM, denatured into single-stranded DNA with 0.1 N NaOH, and subjected to paired-end sequencing on an Illumina MiSeq platform. Raw data were deposited in the NCBI Sequence Read Archive (SRA) under BioProject accession number PRJNA1517325 and will be publicly available upon publication of this article.

For data processing, raw reads were demultiplexed and primers were removed using the demux plugin in QIIME2 (version 2023.5). Quality filtering, denoising, merging, and chimera removal were performed using the DADA2 plugin. The resulting amplicon sequence variants (ASVs) were used for taxonomic assignment against the Greengenes database (version 13_8). Alpha diversity of the gut microbial community was calculated in QIIME2, and differences in alpha diversity indices were assessed using the Kruskal–Wallis test. Beta diversity was evaluated using UniFrac distances and visualized by principal coordinate analysis (PCoA), and between-group differences were tested by permutational multivariate analysis of variance (PERMANOVA). Differentially abundant taxa were identified using the Wilcoxon test and linear discriminant analysis effect size (LEfSe) analysis. The functional potential of the microbiota was predicted using PICRUSt2 (version 2.5.2) against the MetaCyc database. All results were visualized on the GenesCloud platform (https://www.genescloud.cn).

### 2.5. Statistical Analysis

Raw data were compiled and tabulated using WPS 365 (Kingsoft, Beijing, China), and each replicate was considered an independent experimental unit. Before analysis, data normality was assessed using the Shapiro–Wilk test in IBM SPSS Statistics (version 22.0; IBM Corp., Armonk, NY, USA). Intergroup differences were analyzed by one-way analysis of variance (ANOVA), followed by the least significant difference (LSD) test for pairwise comparisons when the ANOVA result was significant. In addition, linear and quadratic polynomial regression models were fitted using the actual allicin dose as a continuous predictor, and the *p*-values for linear and quadratic trends (*P_Linear_* and *P_Quadratic_*) were calculated. Statistical significance was set at *p* < 0.05, and significant differences among groups were indicated by different lowercase letters. Unless otherwise stated, all data are presented as the mean and standard error of the mean.

## 3. Results

### 3.1. Effects of Allicin on Laying Performance

To evaluate the effects of dietary allicin on laying performance, ALR, AEW, ADFI, and FCR were analyzed among the treatment groups (Table 2). Compared with the CON group, AEW was significantly higher in the AL200 and AL400 groups (*p* < 0.05), whereas AEW in the AL600 group was numerically higher but did not differ significantly from the CON group (*p* > 0.05). A significant quadratic trend was detected for AEW with increasing dietary allicin levels (*P_Quadratic_* = 0.001). In addition, ADFI was significantly lower in the AL600 group than in the CON, AL200, and AL400 groups (*p* < 0.05). No significant differences were observed in ALR or FCR among the groups (*p* > 0.05). In summary, dietary allicin supplementation increased AEW at 200–400 mg/kg and reduced ADFI at 600 mg/kg.

### 3.2. Effects of Allicin on Egg Quality

The effects of dietary allicin supplementation on egg quality are presented in Table 3. Compared with the CON group, ESI was significantly higher in all allicin-supplemented groups (*p* < 0.05), and a significant linear trend was observed with increasing dietary allicin levels *(P_Linear_* = 0.020). However, no significant differences were found in YR, AH, HU, or YC among the groups (*p* > 0.05). In summary, dietary allicin supplementation significantly increased ESI in a dose-dependent manner but did not significantly affect YR, AH, HU, or YC.

### 3.3. Effects of Allicin on Lipid Indices

As shown in Table 4, serum TG and LDL-C levels in the AL600 group were significantly lower than those in the CON group (*p* < 0.05), whereas serum TC levels were significantly lower in the AL200, AL400, and AL600 groups than in the CON group (*p* < 0.05). Significant linear trends for decreases in serum TG, TC, and LDL-C were observed with increasing dietary allicin supplementation levels (*P_Linear_* < 0.05). In addition, YTC was significantly lower in the AL400 and AL600 groups than in the CON group (*p* < 0.05), and a significant linear decreasing trend in YTC was detected with increasing allicin supplementation levels (*P_Linear_* = 0.004). No significant differences were observed in serum HDL-C among the groups (*p* > 0.05). In summary, dietary allicin supplementation decreased serum TG, TC, and LDL-C levels and YTC in a dose-dependent manner, with no significant effect on serum HDL-C.

### 3.4. Effects of Allicin on Organ Indices

To evaluate the effects of dietary allicin supplementation on visceral organ indices, hens from the CON and AL600 groups were slaughtered at the end of the experimental period. As shown in Table 5, no significant differences were found in the liver, jejunum, or ovary indices between the AL600 and CON groups (*p* > 0.05). However, AFP was significantly lower in the AL600 group than in the CON group (*p* < 0.05), whereas the duodenal index was significantly higher in the AL600 group than in the CON group (*p* < 0.05). In summary, 600 mg/kg allicin supplementation reduced AFP and increased the duodenal index, without affecting the liver, jejunum, or ovary indices.

### 3.5. Effects of Allicin on Duodenal Microbiota Composition

**Figure 1:**
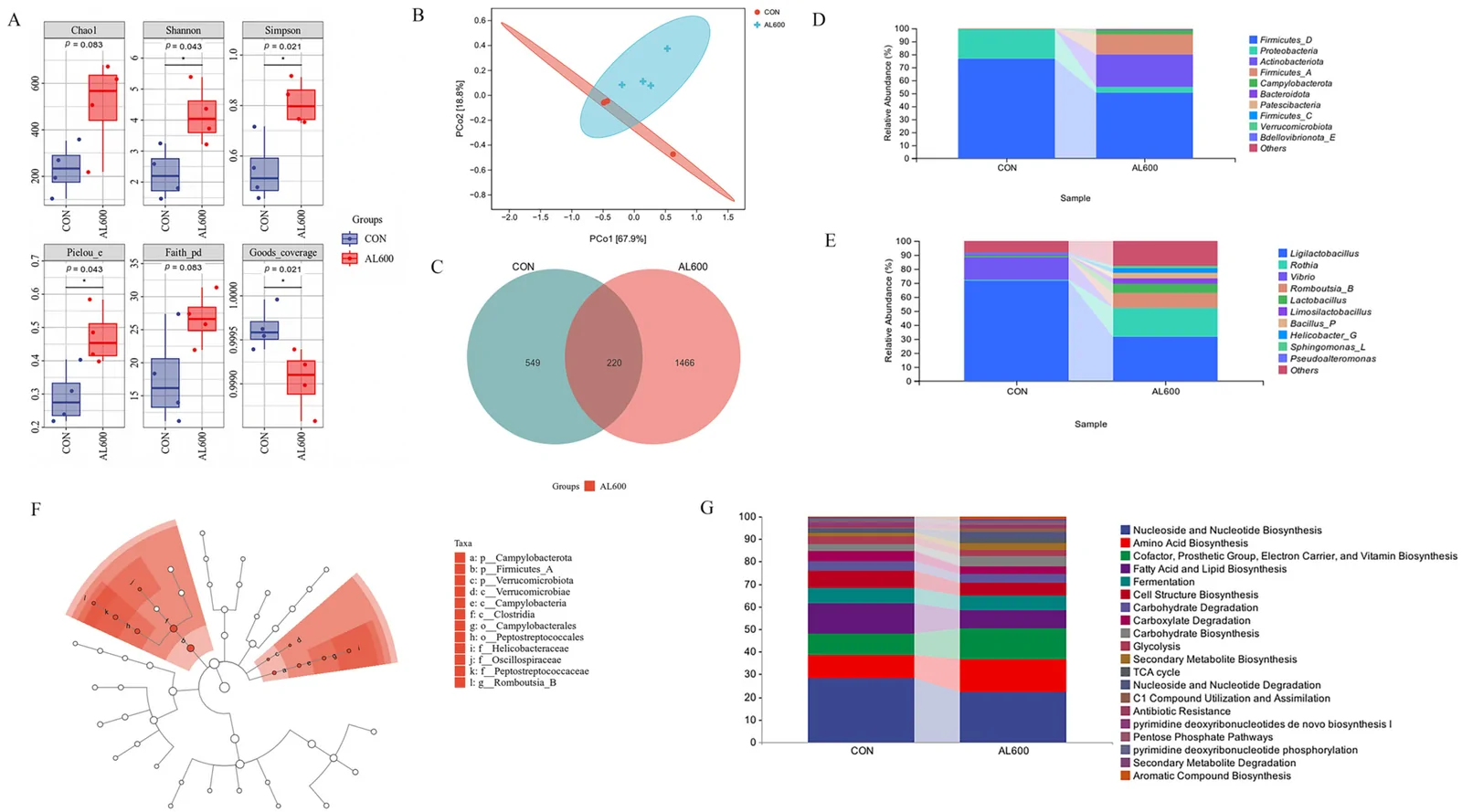
Effects of dietary allicin supplementation on duodenal microbiota composition in Kangle chickens. The CON and AL600 groups received a basal diet supplemented with 0 and 600 mg/kg allicin, respectively. (**A**) Box plots showing alpha diversity indices of the duodenal microbiota in the CON and AL600 groups. (**B**) Principal coordinate analysis (PCoA) plot of microbial community structure in the CON and AL600 groups. (**C**) Venn diagram showing shared and unique amplicon sequence variants (ASVs) between the CON and AL600 groups. (**D**) Phylum-level taxonomic composition showing the top 10 most abundant phyla in the CON and AL600 groups. (**E**) Genus-level taxonomic composition showing the top 10 most abundant genera in the CON and AL600 groups. (**F**) Linear discriminant analysis effect size (LEfSe) cladogram showing differentially abundant taxa between the CON and AL600 groups (*p* < 0.05; LDA score > 4.0). (**G**) Predicted functional composition of the duodenal microbiota based on PICRUSt2 and MetaCyc, showing the top 20 level-2 predicted functional pathways with the highest relative abundance.

To evaluate the effects of dietary allicin supplementation on duodenal microbiota, 16S rRNA gene amplicon sequencing of duodenal contents was performed in the CON and AL600 groups. As shown in Figure 1A, alpha diversity analysis revealed significant differences in the Shannon, Simpson, Pielou’s evenness, and Goods’ coverage indices between the CON and AL600 groups (*p* < 0.05), suggesting that allicin supplementation was associated with alterations in duodenal microbial diversity. In the principal coordinate analysis (PCoA) (Figure 1B), PCo1 and PCo2 together explained 86.7% of the total variance, indicating that the two principal coordinates captured most of the variation. Although PERMANOVA revealed no significant separation between the two groups (*q* = 0.113), the samples within each group showed relatively close clustering, with a trend toward separation between the CON and AL600 groups, suggesting a potential effect of allicin on duodenal microbiota composition.

To further assess differences in microbial community composition between the CON and AL600 groups, Venn diagram and taxonomic composition analyses were performed. The Venn diagram (Figure 1C) identified a total of 2235 ASVs across both groups, of which 549 ASVs were unique to the CON group, 1466 ASVs were unique to the AL600 group, and 220 ASVs (9.84% of the total) were shared between the two groups. These results suggest that dietary allicin supplementation was associated with a greater number of unique ASVs in the duodenum of Kangle chickens.

Taxonomic composition analysis at the phylum level (Figure 1D) showed that *Firmicutes_D, Proteobacteria*, *Actinobacteriota*, and *Firmicutes_A* were the dominant phyla. Their relative abundances in the CON group were 76.70%, 22.56%, 0.45%, and 0.16%, respectively. Compared with the CON group, the AL600 group showed decreases of 26.29 and 18.30 percentage points in the relative abundances of *Firmicutes_D* and *Proteobacteria*, respectively, and increases of 24.78 and 15.51 percentage points in those of *Actinobacteriota* and *Firmicutes_A*, respectively. At the genus level (Figure 1E), *Ligilactobacillus*, *Rothia*, *Vibrio*, and *Romboutsia_B* were the dominant genera. Their relative abundances in the CON group were 71.65%, 0.34%, 16.45%, and 0.01%, respectively, whereas the AL600 group showed decreases of 40.27 and 16.45 percentage points for Ligilactobacillus and Vibrio, respectively, and increases of 20.54 and 10.46 percentage points for *Rothia* and *Romboutsia_B*, respectively.

To identify differential taxa associated with allicin supplementation, LEfSe analysis was performed with thresholds of *p* < 0.05 and LDA score > 4.0, and a cladogram was generated (Figure 1F). Three differential phyla were identified: *Campylobacterota*, *Firmicutes_A*, and *Verrucomicrobiota*. Among these, *Firmicutes_A* was the dominant phylum and exhibited relatively high abundance. Within this phylum, *Clostridia*, *Peptostreptococcales*, *Peptostreptococcaceae*, *Romboutsia_B*, and *Oscillospiraceae* were identified as differential taxa, among them, *Romboutsia_B* was one of the most abundant differential genera. According to LEfSe analysis, the relative abundances of *Firmicutes_A* and its associated differential taxa (*Clostridia*, *Peptostreptococcales*, *Peptostreptococcaceae*, *Romboutsia_B*, and *Oscillospiraceae*) were higher in the AL600 group than in the CON group.

Based on the duodenal microbiota composition, microbial functional potential was predicted using PICRUSt2 against the MetaCyc database, and the top 20 level-2 functional pathways are presented in a bar plot (Figure 1G). Nucleoside and Nucleotide Biosynthesis, Amino Acid Biosynthesis, and Fatty Acid and Lipid Biosynthesis were the most abundant predicted functional pathways. Compared with the CON group (Table S1), the AL600 group had a lower predicted relative abundance of the Fatty Acid and Lipid Biosynthesis pathway, whereas the predicted relative abundances of Carbohydrate Biosynthesis, Secondary Metabolite Biosynthesis, and C1 Compound Utilization and Assimilation pathways were higher. These results suggest that allicin supplementation was associated with shifts in predicted duodenal microbial metabolic functions.

## 4. Discussion

In recent years, plant-derived feed additives have attracted increasing research attention because of the overuse of antibiotics and the associated risk of drug residues in animal products [16]. Allicin, an organosulfur compound derived from garlic, exhibits a wide range of biological effects, including antioxidant, anti-inflammatory, and microbiota-modulating properties [1].

Although progress has been made in investigating the effects of allicin on the laying performance of hens, the reported results remain inconsistent. Several studies have found that allicin and its related products have no or only minor effects on laying performance [13,17,18]. However, Adjei-Mensah et al. [19] reported that supplementation of drinking water with 1.0 mL/L garlic extract, which contains allicin and its derivatives as its main active constituents, significantly increased egg weight. The present results are broadly consistent with these observations, although the response appeared to depend on the supplementation level. The increase in egg weight may be attributed to improved nutrient utilization and intestinal health associated with appropriate allicin supplementation [1], which could enhance the availability of proteins and lipids for yolk deposition. At higher supplementation levels, however, these potential benefits may be attenuated or offset by reduced feed intake due to the irritant properties of high-dose allicin, which could in turn impair egg weight. ESI is an important quality trait associated with commercial egg grading standards [20]. The increase in ESI observed in the present study may have practical implications for egg production. ESI is influenced by eggshell formation, calcium metabolism, and oviduct function. Allicin may improve ESI indirectly through its antioxidant and microbiota-modulating effects [8,12]; however, direct evidence for this mechanism in laying hens is lacking, and the exact mechanism remains to be elucidated. Given the variation in dosage, supplementation form, animal breed, and experimental duration among studies, the effects of allicin on laying performance remain inconsistent, warranting further investigation.

Allicin and its derivatives have been reported to regulate serum lipid metabolism in chickens. Previous studies have shown that dietary supplementation with 7.5 g/kg garlic powder significantly reduced TG and LDL-C levels in broilers [8], and that TC levels decreased significantly with increasing dietary garlic powder inclusion (0.5%, 1.0%, and 1.5%) [21]. Furthermore, Amiri et al. [22] reported that supplementation with 75 mg/kg nano-encapsulated garlic essential oil (GEO) significantly reduced serum TC and LDL-C concentrations in chickens, indicating that different garlic preparations can regulate lipid metabolism in chickens. In the present study, dietary allicin significantly decreased serum TC and LDL-C levels in Kangle chickens in a dose-dependent manner, consistent with the above findings. However, only supplementation with 600 mg/kg allicin significantly reduced serum TG levels in the present study. This discrepancy in the effective dose may be attributable to differences in supplementation duration, administration route, and animal breed among studies.

Allicin regulates lipid metabolism through multiple mechanisms. It has been reported that allicin directly reduces endogenous cholesterol and fatty acid synthesis by inhibiting the activities of rate-limiting enzymes involved in cholesterol synthesis, such as HMGCR and squalene epoxidase (SQLE), and by suppressing the expression of key lipogenic regulators, including sterol regulatory element-binding protein 1c (SREBP1c) and FAS [23,24,25]. In addition, allicin interferes with intestinal lipid absorption and transport by downregulating microsomal triglyceride transfer protein (MTP) expression [26]. Moreover, allicin promotes reverse cholesterol transport from peripheral cells by activating the PPARγ–LXRα signaling pathway and upregulating *ATP-binding cassette subfamily A member 1* (*ABCA1*) gene expression [27]. It also induces the expression of cholesterol 7α-hydroxylase (CYP7A1), a key enzyme in bile acid metabolism, thereby accelerating the conversion of cholesterol to bile acids [27]. Furthermore, allicin modulates gut microbiota, and this modulation may influence lipid metabolism through the gut–liver axis by increasing the abundance of beneficial bacteria such as Bifidobacterium and Lactobacillus [28]. Collectively, allicin regulates the synthesis, transport, and efflux of cholesterol and other lipids through multiple pathways, thereby lowering serum lipid levels.

Cholesterol is an essential component and nutrient in animals and plays a crucial role in maintaining metabolic homeostasis [29]. However, excessive cholesterol intake contributes to the development of various human diseases [30], leading to growing public concern regarding dietary cholesterol content. Therefore, developing effective nutritional strategies to reduce yolk cholesterol content is of considerable interest for the laying hen industry. Allicin has attracted increasing attention for its potential to regulate cholesterol metabolism and reduce cholesterol deposition. Chowdhury et al. [31] reported that dietary supplementation with 2% to 10% garlic paste for six consecutive weeks significantly reduced yolk cholesterol content in 28-week-old laying hens from six strains. Similarly, Yalçin et al. [17] found that dietary supplementation with 10 g/kg garlic powder for 22 consecutive weeks significantly decreased yolk cholesterol content in laying hens. Consistent with these findings, the present study demonstrated that dietary supplementation with an appropriate level of allicin significantly reduced yolk cholesterol content in Kangle laying hens. As described above, allicin regulates lipid metabolism through multiple pathways, including suppression of cholesterol synthesis, interference with intestinal lipid absorption and transport, promotion of reverse cholesterol transport, and modulation of gut microbiota. These mechanisms may collectively reduce the hepatic cholesterol pool available for yolk deposition, thereby contributing to the lower yolk cholesterol content observed in this study. These findings suggest that dietary allicin supplementation may serve as a natural and effective strategy for reducing yolk cholesterol levels, although an appropriate supplementation level is required.

The gut microbiota is often regarded as an “invisible organ” of the host and plays critical roles in nutritional synthesis, metabolic transformation, immune modulation, and intestinal defense [32,33,34]. Allicin has been shown to modulate gut microbiota. Studies have demonstrated that dietary supplementation with garlic oil or garlic extract significantly reduced the abundance of potentially harmful bacteria, such as *Escherichia coli* and *Salmonella*, in the small intestine while increasing the abundance of potentially beneficial bacteria, including *Lactobacillus* and *Enterococcus*, thereby improving intestinal morphology [35] and immune function [9,35,36]. Furthermore, allicin can modulate host metabolic functions by altering microbial community structure. For instance, dietary supplementation with 0.5% allicin combined with α-ketoglutarate in growing pigs significantly increased cecal microbial richness and diversity [37] and enhanced short-chain fatty acid (SCFA) production [38,39,40].

The allicin used in the present study is a volatile organosulfur compound that is primarily absorbed in the duodenum and upper small intestine [41,42]. The microbial community in this intestinal segment exhibits distinct characteristics compared with other gut regions [43,44,45], and its structure is highly sensitive to the host metabolic state [46]. Therefore, the duodenum was selected as the target intestinal segment in this study. The results showed significant differences between the CON and AL600 groups in the Shannon, Simpson, and Pielou’s evenness indices. The Shannon index is particularly sensitive to rare species [47], whereas the Simpson index reflects the dominance of abundant species within a community [48]. The simultaneous increases in both indices suggest that allicin may have contributed to improved duodenal microecological health. Pielou’s evenness measures the evenness of species relative abundance distribution independent of species richness [49,50], and higher Pielou’s evenness values generally indicate greater stability and resilience of the gut microbial ecosystem against perturbations [51]. The increases in these diversity indices suggest that allicin may modulate duodenal microbial community composition and contribute to intestinal homeostasis in Kangle chickens, which is generally consistent with previous findings.

*Proteobacteria* is a major phylum of Gram-negative bacteria that includes various opportunistic pathogens [52] and has been closely associated with intestinal inflammation and metabolic disorders [52,53,54]. Therefore, the numerically lower relative abundance of this phylum, together with the lower relative abundance of *Vibrio* (a genus within *Proteobacteria*), may indicate that allicin is associated with amelioration of microbial dysbiosis, particularly at higher supplementation levels. *Firmicutes_A* and its related taxa, including *Clostridia*, *Peptostreptococcales*, *Peptostreptococcaceae*, *Romboutsia_B*, and *Oscillospiraceae*, are known to participate in host immune regulation and intestinal barrier maintenance. Members of *Oscillospiraceae* can degrade mucin glycoproteins to release oligosaccharides and SCFAs, which not only provide energy substrates for epithelial cells but also promote intestinal stem cell proliferation and goblet cell differentiation, thereby accelerating epithelial regeneration and enhancing tight junction protein expression to maintain mucosal barrier integrity [55,56]. Furthermore, *Oscillospiraceae* is recognized as a butyrate-producing taxon and may increase butyrate availability, thereby enhancing intestinal epithelial barrier function and alleviating local inflammatory responses, which is important for maintaining gut health [57]. The higher relative abundance of these microbial taxa may therefore contribute to improved intestinal function, which is generally consistent with the improved laying performance of Kangle chickens.

The differential taxa identified in the present study may also play roles in lipid metabolism and cholesterol homeostasis. Studies have demonstrated that metabolites of the genus *Romboutsia*, particularly *Romboutsia ilealis*, such as 2-oxoindole-3-acetate (OAA), directly inhibit host intestinal lipid absorption and alleviate obesity through the OAA–PSMD3–YTHDF2–Rxrb signaling axis [58]. In addition, *Romboutsia* contributes to the reduction of oxidized lipids and promotes browning of white adipose tissue, and its abundance is negatively correlated with oxidized lipid levels [59]. *Oscillospiraceae* is an important bile acid-converting microbial group in the intestine that modulates bile acid pool composition through 7α-dehydroxylation, promotes cholesterol excretion, and thereby influences host cholesterol levels [60,61]. Studies have shown that the abundance of *Oscillospira* is negatively correlated with host serum lipid levels and obesity indices, suggesting that it may have a protective role in maintaining lipid metabolic homeostasis [62]. Certain members of the class *Clostridia* are also involved in bile acid conversion and SCFA production, indirectly regulating lipid metabolism [63]. These microbial functions may therefore contribute to the improved lipid metabolic profile associated with allicin supplementation in Kangle chickens, which is consistent with the lipid-lowering effects observed in this study.

However, several limitations should be considered when interpreting the effects of dietary allicin supplementation on laying performance, egg quality, serum lipid indices, and duodenal microbiota in Kangle chickens. First, only one indigenous chicken breed (Kangle chickens) was used, and the experimental period was relatively short. In addition, the duodenal microbiota analysis was limited because sequencing was performed only for the CON and AL600 groups, and the sample size was relatively small. Therefore, the microbiota results should be interpreted with caution and considered exploratory. Future studies involving multiple indigenous breeds, larger sample sizes, longer experimental periods, and microbiota sequencing of all treatment groups are needed to further verify the effects of allicin in Kangle chickens and other indigenous chicken breeds.

## 5. Conclusions

Collectively, dietary allicin supplementation increased average egg weight at 200–400 mg/kg, improved egg shape index at all tested levels, and reduced serum total cholesterol levels in Kangle laying hens. Supplementation at 400–600 mg/kg also reduced yolk total cholesterol content. In addition, supplementation with 600 mg/kg allicin was associated with shifts in duodenal microbiota composition and predicted metabolic functions. These findings provide a scientific reference for the application of allicin as a feed additive in Kangle chickens and may inform its use in other indigenous chicken breeds. The results suggest that 400 mg/kg allicin may be an appropriate supplementation level for improving egg quality and reducing cholesterol levels. Further studies are needed to confirm the optimal supplementation level and to elucidate the underlying mechanisms.

## Figures and Tables

**Table 1:** Ingredients and nutrient components of basal diets (air-dry basis).

Ingredients	Content (%)	Nutrient Components	Content (%) ^2^
Maize	58.5	Metabolizable energy (MJ/kg)	11.47
Soybean meal	8.90	Crude protein	16.63
Corn gluten meal	7.00	Lysine	0.78
Wheat bran	4.50	Methionine + Cystine	0.68
Soybean oil	1.86	Calcium	3.50
Rapeseed meal	7.50	Available phosphorus	0.34
Lysine	0.25		
Methionine	0.07		
Dicalcium phosphate	1.40		
Limestone	8.60		
Choline chloride	0.10		
Phytase	0.02		
Salt	0.30		
Premix ^1^	1.00		
Total	100		

^1^ The premix provided the following per kilogram of the diet: Fe 60 mg, Cu 8 mg, Zn 80 mg, Se 0.30 mg, Mn 60 mg, I 0.35 mg, VA 8000 IU, VD_3_ 1600 IU, VE 3.34 mg, VK 0.50 mg, thiamine 0.80 mg, niacin 20 mg, riboflavin 2.5 mg, folic acid 0.25 mg, calcium pantothenate 2.20 mg, tocopherol 0.10 mg, pyridoxine 3.00 mg, and cobalamin 0.004 mg. All nutrient contents were calculated values; ^2^ The metabolizable energy (ME) was estimated from near-infrared spectroscopy (NIRS)-derived feed composition, following Cozannet et al. [15].

**Table 2:** Effects of dietary allicin supplementation on laying performance of Kangle chickens.

Item ^1^	Treatments ^2^				SEM	*p*-Value	*P_Linear_*	*P*_Quadratic_
	CON	AL200	AL400	AL600				
ALR (%)	63.33	60.6	57.62	59.52	0.96	0.203	0.197	0.255
AEW (g)	41.78 ^b^	43.18 ^a^	43.38 ^a^	41.92 ^b^	0.13	0.001	0.595	0.001
ADFI (g/d)	83.52 ^a^	85.86 ^a^	82.64 ^a^	75.04 ^b^	0.95	0.001	0.001	0.001
FCR	2.78	2.74	2.73	2.59	0.04	0.410	0.142	0.053

^1^ ALR: average laying rate; AEW: average egg weight; ADFI: average daily feed intake; FCR: feed conversion ratio. ^2^ The CON, AL200, AL400, and AL600 groups were supplemented with 0, 200, 400, and 600 mg/kg allicin, respectively. Values within the same row with different lowercase letter superscripts indicate significant differences (*p* < 0.05). *P_Linear_* and *P_Q__uadratic_* indicate the significance of the linear and quadratic terms, respectively.

**Table 3:** Effects of dietary allicin supplementation on egg quality of Kangle chickens.

Items ^1^	Treatments ^2^				SEM	*p*-Value	*P_Linear_*	*P*_Quadratic_
	CON	AL200	AL400	AL600				
YR	0.30	0.31	0.30	0.30	0.01	0.749	0.514	0.810
ESI	1.26 ^b^	1.30 ^a^	1.32 ^a^	1.31 ^a^	0.01	0.005	0.020	0.058
AH (mm)	5.16	5.37	5.23	5.04	0.10	0.598	0.850	0.327
HU	76.65	78.56	76.04	76.23	0.72	0.507	0.878	0.425
YC	9.33	8.87	8.67	9.33	0.13	0.124	0.635	0.134

^1^ YR: yolk ratio; ESI: egg shape index; AH: albumen height; HU: Haugh unit; YC: yolk color. ^2^ The CON, AL200, AL400, and AL600 groups were supplemented with 0, 200, 400, and 600 mg/kg allicin, respectively. Values within the same row with different lowercase letter superscripts indicate significant differences (*p* < 0.05). *P_Linear_* and *P_Q__uadratic_* indicate the significance of the linear and quadratic terms, respectively.

**Table 4:** Effects of dietary allicin supplementation on lipid parameters of Kangle chickens.

Items ^1^	Treatments ^2^				SEM	*p*-Value	*P_Linear_*	*P*_Quadratic_
	CON	AL200	AL400	AL600				
TG (mmol/L)	4.57 ^a^	3.72 ^a^	3.36 ^a^	2.37 ^b^	0.24	0.045	0.005	0.020
TC (mmol/L)	3.57 ^a^	2.82 ^b^	2.85 ^b^	2.60 ^b^	0.13	0.012	0.002	0.006
HDL-C (mmol/L)	0.88	0.96	0.92	0.66	0.04	0.196	0.157	0.093
LDL-C (mmol/L)	1.46 ^a^	1.14 ^a^	1.33 ^a^	0.56 ^b^	0.10	0.002	0.003	0.006
YTC (μmol/g)	25.8 ^a^	20.63 ^ab^	20.39 ^b^	17.03 ^b^	0.96	0.023	0.004	0.018

^1^ TG: triglyceride; TC: total cholesterol; HDL-C: high-density lipoprotein cholesterol; LDL-C: low-density lipoprotein cholesterol; YTC: yolk total cholesterol. ^2^ The CON, AL200, AL400, and AL600 groups were supplemented with 0, 200, 400, and 600 mg/kg allicin, respectively. Values within the same row with different lowercase letter superscripts indicate significant differences (*p* < 0.05). *P_Linear_* and *P_Q__uadratic_* indicate the significance of the linear and quadratic terms, respectively.

**Table 5:** Effects of dietary allicin supplementation on organ indices of Kangle chickens.

Items ^1^	Treatments ^2^		SEM	*p*-Value
	CON	AL600		
Liver index (%)	1.82	1.86	0.08	0.770
Duodenal index (%)	0.72	0.81	0.02	0.029
Jejunum index (%)	1.90	2.07	0.06	0.115
Ovary index (%)	0.56	0.53	0.03	0.678
AFP (%)	3.89	2.61	0.25	0.004

^1^ AFP: abdominal fat percentage. ^2^ The CON and AL600 groups were supplemented with 0 and 600 mg/kg allicin, respectively.

## Data Availability

The data presented in this study have been deposited in the NCBI Sequence Read Archive (SRA) under accession number PRJNA1517325 and will be publicly available upon publication of this article.

## Supplementary Materials

The following supporting information can be downloaded at: https://www.mdpi.com/article/10.3390/ani16203168/s1, Table S1: The results of differential analysis of microbial metabolic pathways.
