# Supplementary material for: Effects of Dietary Allicin Supplementation on Laying Performance, Egg Quality, Serum Lipid Indices, and Gut Microbiota Composition in Kangle Chickens
Source: Animals (Basel). 2026 Oct 9;16(20):3168. doi: 10.3390/ani16203168 (PMC13700906; doi:10.3390/ani16203168)
Supplement: Supplementary file 1 [file animals-16-03168-s001.zip › animals-4518361-supplementary.pdf]

Table S1. The results of differential analysis of microbial metabolic pathways

Items[1]	Treatments[2]		SEM	*P*-value
	**CON**	**AL600**		
Nucleoside and Nucleotide Biosynthesis	46424.62	43270.19	2147.55	0.641
Amino Acid Biosynthesis	16305.02	28215.00	2840.64	0.149
Cofactor, Prosthetic Group, Electron Carrier, and Vitamin Biosynthesis	15782.56	26858.11	3658.68	0.320
Fatty Acid and Lipid Biosynthesis	22111.76	15732.71	1119.52	0.028
Fermentation	11203.59	12849.39	578.49	0.353
Cell Structure Biosynthesis	12437.21	10880.95	509.53	0.315
Carbohydrate Degradation	7123.01	7207.22	377.56	0.944
Carboxylate Degradation	7143.25	6902.33	380.94	0.842
Carbohydrate Biosynthesis	4793.80	8846.81	788.73	0.060
Glycolysis	5808.12	5331.61	341.90	0.659
Secondary Metabolite Biosynthesis	2370.13	5810.89	536.52	0.007
TCA cycle	1716.24	4995.20	821.02	0.173
Nucleoside and Nucleotide Degradation	1740.32	4865.17	754.42	0.155
C1 Compound Utilization and Assimilation	749.34	2958.96	355.72	0.011
Antibiotic Resistance	1869.82	1545.94	127.73	0.412
pyrimidine deoxyribonucleotides de novo biosynthesis I	1882.05	1473.28	154.77	0.391
Pentose Phosphate Pathways	789.02	2367.93	308.44	0.061
pyrimidine deoxyribonucleotide phosphorylation	1751.54	1353.48	116.81	0.257
Secondary Metabolite Degradation	926.06	1891.72	283.20	0.257
Aromatic Compound Biosynthesis	630.87	1880.26	270.79	0.104

[1] Based on the MetaCyc database. [2] The CON and AL600 groups were supplemented with 0 and 600 mg/kg allicin, respectively.
